# Addressing External Shock in Urban Agglomeration: Implications From the Transmission Pattern of COVID-19 in the Beijing-Tianjin-Hebei Area

**DOI:** 10.3389/fpubh.2022.870214

**Published:** 2022-05-11

**Authors:** Daohan Huang, Fenghua Wen, Shunru Li

**Affiliations:** ^1^School of Urban Economics and Management, Beijing University of Civil Engineering and Architecture, Beijing, China; ^2^School of Government, Central University of Finance and Economics, Beijing, China

**Keywords:** COVID-19 epidemic, risk prevention and control, conceptual framework, comprehensive risk, Beijing-Tianjin-Hebei area

## Abstract

Properly addressing external shocks in urban agglomeration is critical to sustaining the complex regional system. The COVID-19 pandemic has been widely acknowledged as an unintended external shock, but the temporal and spatial transmission patterns are largely ignored. This study analyzed the temporal and spatial transmission patterns of COVID-19 at the macro, meso, and micro levels, and proposes a conceptual model for regional comprehensive risk calculation, taking the Beijing-Tianjin-Hebei (BTH) area as the focus region. Our results showed that 1) at the temporal scale, the epidemic in the BTH area experienced stages of rapid increase, gradual decrease, and stabilization, and the first wave of the epidemic was under control from 23 February 2020; 2) at the spatial scale, confirmed cases were largely distributed at the terminal of the migration network, with closely interconnected cities in the BTH area, including Beijing, Tianjin, Tangshan, and Langfang, holding the highest comprehensive epidemic risk, thus requiring special attention for epidemic prevention and control. Finally, a “two-wheels” conceptual framework was built to discuss implications for future policies for addressing external shocks. Our proposed framework consists of an isolation wheel, which involves information sharing from the holistic perspective, and a circulation wheel, which emphasizes stakeholder involvement from the individual perspective. The findings of this study provide a knowledge basis for epidemic prevention and control as well as useful implications for addressing external shocks in the future.

## Introduction

Urban agglomeration integrates adjacent cities through infrastructures, supply chains, and transferred industries (1). These integrated cities form a spatial network at the regional level, sharing regional land resources and environmental capacity, which are important to accelerate local economy growth (2). Although co-dependence in urban agglomeration can enhance the ability of single cities to address internal and external shocks (3, 4), the risk is also transferred *via* interconnections between integrated cities, resulting in significant unintended consequences. This indicates that a small internal or external shock may induce a large crisis at the regional scale in the urban agglomeration, which will get enhanced in the current risk society (5). Therefore, avoiding a crisis in addressing internal and external shocks is a critical step to achieving a sustainable region system in urban agglomeration. Taking COVID-19 as the external shock, we aimed to explore the infection and transmission patterns of COVID-19 in the Beijing-Tianjin-Hebei (BTH) area.

COVID-19 was declared a pandemic by the World Health Organization in March 2020. Mask wearing, isolation, and vaccination are effective measures to mitigate the COVID-19 pandemic. Although the world is experiencing a new huge wave of infection with the omicron variant recently, the end of the pandemic could be near (6). The past 2 years of the COVID-19 pandemic can be divided into three phases, that is, the panic, adjustment, and coexistence phases (7, 8). China has experienced these three phases since the end of December 2019, when a COVID-19 outbreak first occurred in Wuhan. The panic phase in China lasted from January to March 2020, during which a containment strategy was adopted by the government of China (9). This strategy was able to eradicate the epidemic in mainland China but exerted a negative influence on the social and economic activities. For example, COVID-19 had significant risk spillover effects on Chinese energy sectors; in fact, the oil exploitation sector held the highest and longest volatility spillover during the panic phase (10). Next, the adjustment phase in China occurred between April 2020 and July 2021, during which a mitigation strategy was adopted (11). The focus of this strategy was to block the imported epidemic and control the local epidemic with a large-scale nucleic acid test. Finally, in the coexistence phase, the dynamic COVID-zero strategy has been conducted since August 2021. To live with COVID-19, precision prevention measures are implemented on the whole cycle of COVID-19 transmission to control the pandemic at the lowest cost. In the coexistence phase, a 20-m^2^ milk tea store in Shanghai became the smallest medium-risk area but did not pose a spillover risk to the neighboring store. Therefore, this study focused on the panic phase in the BTH area, as COVID-19 in this phase was treated as an unpredictable external shock.

COVID-19 in the panic phase has been widely regarded as a perfect example of external shock in studies aiming to explore the impacts of this disease on the regional economy (12) and environment (13). On the city scale, the COVID-19 shock affected urban landscape planning, compact city, and resource supply chain (14–16), which requires rethinking of health implications in the post-pandemic era. This kind of research provides insight into the influence pattern of the COVID-19 shock, along with useful strategies to alleviate the negative impacts. For example, the current studies have concluded that the COVID-19 shock exerts negative effects on the economy and normal life, which can be alleviated by a digitalization strategy (13, 17). From the perspective of economic geography, this is because the isolation requirement among workers during the pandemic contradicts the circulation demands in economic development (18). However, the spatial pattern of the epidemic transmission has been largely ignored in the current studies, which could provide a knowledge basis on the infection patterns and implications. Liu et al. (19) employed the bottom-up approach to explore the epidemic transmission mechanism and its characteristics in the Guangdong Province. Li et al. (20) and Li et al. (21) analyzed COVID-19 transmission in the Shanxi Province, Tianjin City, and Chongqing City *via* a top-down analysis with a focus on imported cases. These studies focused on intra-provincial rather than inter-provincial agglomeration, and the top-down analysis was inefficient because it centered on the temporal scale. Therefore, in this study, we conducted a top-down analysis of the spatial pattern of COVID-19 transmission in the inter-provincial BTH area.

A total of 13 cities are integrated into the BTH area, including two megacities, Beijing and Tianjin, making it one of the four national urban agglomeration areas. The BTH area is facing serious external shocks, especially environmental risks such as local water shortage and air pollution (3). Thus, cooperation mechanisms to face the external shock are being implemented at the provincial government level. As the capital of China, Beijing always takes the strictest measures in addressing external shocks for security purposes. This largely limits the circulation of production elements and workforces between cities during the pandemic. For example, the group commute from Hebei or Tianjin to Beijing increases their time cost with strict personal checks in the panic phase, which require an effective cooperation mechanism between cities. To this end, the BTH area is an ideal agglomeration area for studying the infection pattern of COVID-19.

To address the above gaps, we conducted a theoretical analysis of the infection and transmission patterns of COVID-19 in the Methodology and theoretical analysis section. Next, taking the BTH area as an example, we studied the spatial and temporal characteristics of the COVID-19 epidemic in the panic phase and assessed the transmission risk in the Results analysis section. Future implications on the measures to address external shocks are discussed in the Discussions section, followed by the Conclusions section. The findings of this will provide a knowledge basis for epidemic prevention and control as well as useful insights for addressing external shocks in the future.

## Methodology and Theoretical Analysis

Infectious diseases are caused by pathogenic microorganisms (e.g., viruses, bacteria, and fungi) and parasites (e.g., protozoa and helminths); they can infect susceptible populations and may result in epidemics under certain conditions (22). A sudden infectious disease refers to an infectious disease that has no evident symptoms and no evident seasonal or regional patterns, and can easily break out on a large scale and spread rapidly among the population, causing serious negative impacts on the economy and society (23). The transmission of infectious diseases requires three conditions, that is, an infection source, a transmission route, and a susceptible population. COVID-19 is a sudden infectious disease caused by the novel coronavirus, which infects susceptible populations *via* direct (person-to-person contact and surface-to-person contact), aerosol, and contact transmission (24, 25).

**Figure 1:**
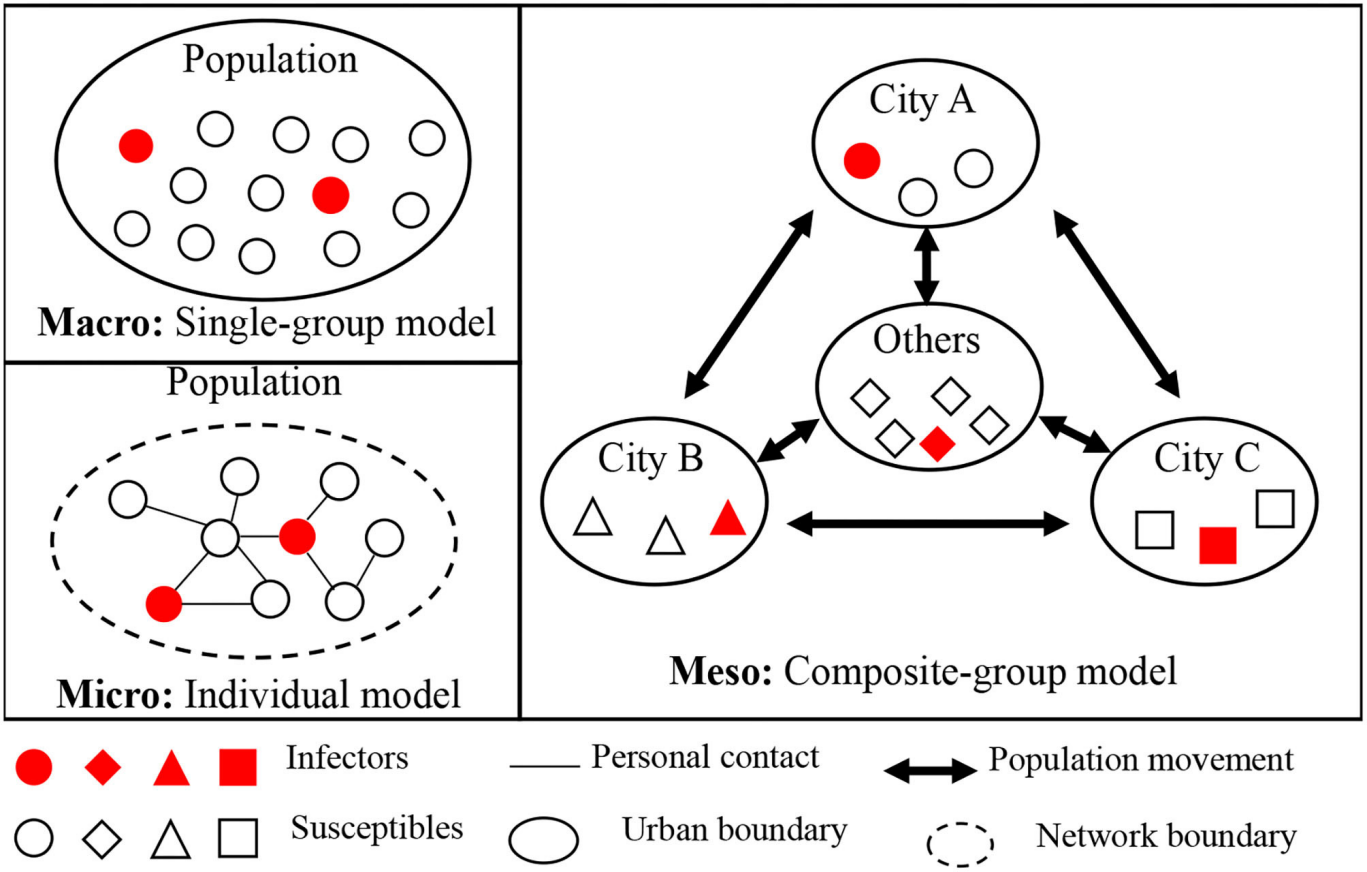
Conceptual framework of regional epidemic transmission patterns.

The effective prevention and control of public health emergencies require dynamic decision-making based on the epidemic development in which scientific exploration of the infection pattern is a critical step. Numerous theoretical and empirical studies focusing on transmission dynamics have been conducted (26). Several studies mapped the life cycle evolution of infectious diseases in China using descriptive epidemiological (27) and statistical methods (28, 29). These studies provide a basis for the development of effective prevention and control measures at the national level. Other studies focused on modeling infectious diseases. Theoretically, infectious disease transmission can be summarized into three basic models, namely the single-group model, composite-group model, and individual model [(30); Figure 1 Conceptual framework of regional epidemic transmission patterns]. Zhang et al. (31) summarized the characteristics as well as data and application requirements of the three basic models and further analyzed the most effective model for epidemic prediction. The single-group model is a macro modeling work (Macro: single-group model) highlighting the holistic variation of the susceptible population. Assuming that everybody in the susceptible population has the same susceptible condition, the transmission process can be described as the change in the number of infected, exposed, hospitalized, and recovered individuals. At the meso level, the composite-group model (Meso: composite-group model) considers spatial agglomeration differences in a susceptible population and focuses on interactions between subgroups. The whole susceptible population is divided into several subgroups based on their locations or social space, and the interconnections between the divided subgroups are defined by personal movement. For example, the personal movement between Hubei and other provinces led to the spread of COVID-19 (20). Finally, the micro individual model (Micro: individual model) takes the assumption that individuals have their transmission networks, but everybody is in the same susceptible condition. The contact between an infector and a susceptible individual allows the transmission of an infectious disease. The micro modeling work focuses on personal contact networks and individual behaviors. Many practical models based on the three basic models have been developed to explore transmission dynamics, such as the warehouse, network, and multi-network models (23, 27); transmission dynamics model (32, 33); and differential equation model (21, 34). These practical models quantitatively explore the spatial-temporal transmission patterns of infectious diseases, evaluate the effectiveness of control measures, and predict the future course of an epidemic (34, 35).

The regional epidemic transmission patterns of COVID-19 can be further explained at the macro, meso, and micro levels.

At the macro level, the transmission of the disease is caused by large-scale gatherings of the susceptible population, such as the 10,000-family dinners held in Wuhan on 18 January 2020, and the 10,000-people marathon held in Los Angeles on 9 March 2020. The large-scale aggregation leads to the boundary expansion of the personal contact network and exposes the susceptible population to infectors at a specific time and space. This transmission pattern can result in a widespread epidemic. Therefore, as macro-level prevention and control measures, large-scale population gatherings activities should not be held, and movement of the susceptible population should be reduced, which has been included in the clinical guideline of China (36). In the panic phase, detailed information on newly infected cases, suspected cases, and deaths in the region, as well as the movement tracks of confirmed cases, has been provided in the regional epidemic bulletin. Information on this local infection pattern not only helps the public understand the development of the epidemic and their contact with the epidemic but also minimizes the impact of fake news with authoritative information (37). In China, the reporting of the progress of infectious diseases is localized, which is an easy duty to fulfill but may lead to the lack of reporting at the regional level, such as in the BTH area, and failure to characterize the regional transmission patterns at the macro level.

At the meso level, epidemic transmission occurs through goods-to-person and person-to-person transmission links owing to the movement of infectors between cities. This indicates that the prevention and control of the virus depend on local governments (38). Goods-to-person transmission occurs in frozen food supply chains, such as in the outbreak in Beijing in June 2020 and January 2022. Thus, daily nucleic acid tests and closed-loop management are required for all related workers to prevent the transport of imported cases (39). To prevent person-to-person transmission, specific measures to limit inter-city movement should be taken. First, the susceptible population should be divided into different subgroups by administrative boundaries, such as province, city, county, street, and community. This division emphasizes the territorial duty in controlling the movement of each subgroup. Furthermore, the extension of the Spring Festival holiday and the implementation of online courses in colleges and universities can largely reduce population movement at the provincial level. Finally, immediate lockdown of communities with infectors or severely infected districts is key to reducing the spread of imported cases (21, 34).

At the micro level, epidemic transmission occurs in personal contact networks in which infectors are connected. The epidemic will then spread within the individual contact network. Thus, contact tracing is the most effective approach to reducing transmission risk (21, 40), which is still implemented in China. To maximize the contact network boundary, the first step is to interview the infectors and publish information on their movement, and the micro individual approach should be adopted to identify the infected cases. In the next step, individuals within the contact network boundaries should be isolated for 14 days in the panic phase. The requirements to wear a mask, scan personal health codes, and check personal travel routes in public places are adopted to improve the efficiency of defining contact networks. These measures are helpful to reduce transmission in the contact networks.

Based on these macro-, meso-, and micro-level epidemic transmission patterns, the movement of the susceptible population at the meso and micro scales exerts a positive effect on the spatial and temporal population aggregation at the macro scale. Thus, the conceptual model of comprehensive epidemic risk can be used to integrate these transmission patterns into the basic and expansion coefficient dimensions. The basic coefficient dimension refers to the objective comprehensive epidemic risk of a region. For example, the comprehensive epidemic risk of Wuhan in the Hubei Province is higher than that of the other cities in China, as Wuhan was the eye of the storm in the panic phase. This is usually represented by the infection rate. The expansion coefficient dimension emphasizes the positive effect of external population migration and internal personal movement on epidemic transmission. This is indicated by the passive infection risk caused by external population migration and the active infection risk caused by local personal movement. Therefore, the mathematical expression of the conceptual model of regional epidemic comprehensive risk can be obtained, as shown in Equation (1).


(1) $$\begin{array}{l} \text{C}{\text{R}}_{\text{i}}=\alpha_{\text{i}}*\text{B}{\text{R}}_{\text{i}} \end{array}$$

where CR stands for comprehensive risk; α represents the expansion coefficient; BR is the basic coefficient; and i (= 1, 2, 3,...) is the subgroup code.

## Results Analysis

According to the public data from the National Health Commission, Beijing announced the first confirmed imported COVID-19 case on 20 January 2020. Since then, 1,077 cases had been confirmed in the BTH region until 31 March 2020, including 870 local cases and 207 imported cases. Local transmission of COVID-19 had been controlled since 29 February 2020, and the imported cases had not induced local transmission at that point. This section discusses the accumulative total cases confirmed from 20 January to 29 February 2020. The case data were collected from the official epidemic bulletin published daily by the Health Commission of Beijing, Tianjin, and Hebei, which can be found in the special epidemic column on their official website. Policies were collected from the national and local Health Commission departments. The analysis was conducted at the macro, meso, and micro levels, followed by the calculation of comprehensive risk using our proposed conceptual framework.

### Macro Transmission Pattern: Epidemic Transmission Trend in the BTH Area

**Figure 2:**
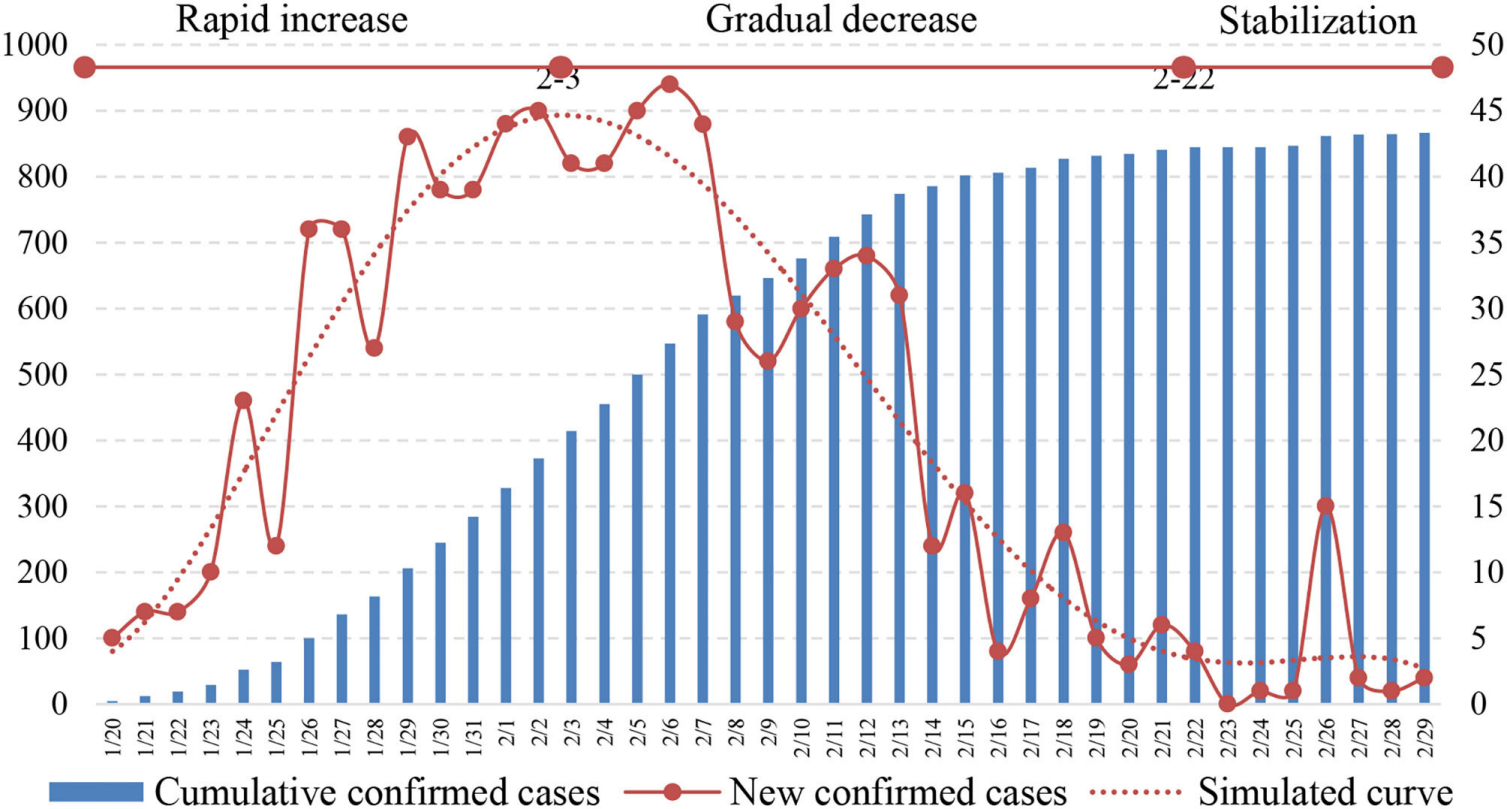
Number of new and cumulative confirmed cases.

The three stages of COVID-19 transmission in the BTH area are specified, that is, rapid increase, gradual decrease, and stabilization. The epidemic in the BTH area started on 20 January 2020, when the first patient was diagnosed. Based on the analysis of new confirmed cases and cumulative confirmed cases, the evolution of the epidemic in the BTH area can be summarized into three stages according to the number of new confirmed cases, namely the rapid increase (20 January−3 February), gradual decrease (4 February−22 February), and stabilization (23 February−29 February) as shown in Figure 2.

In the rapid increase phase, the number of new confirmed cases increased quickly, and the cumulative number of confirmed cases was still small. This indicated that the epidemic was in the outbreak stage. Results showed that this period lasted for 14 days, which is consistent with the incubation period of COVID-19 (39). Moreover, the early imported cases are fully confirmed during this stage, which leads to the increasing trend. The other reason is the response from the local government, such as contact tracing as well as improved diagnostic criteria and nucleic acid detection capabilities. On 3 February 2020, the average number of new confirmed cases gradually stabilized at 45 cases per day and then began to fluctuate. In the gradual decrease stage of the epidemic, the average number of new confirmed cases decreased from 45 to 5 cases per day, and the cumulative number of confirmed cases increased with a decreasing growth rate. In the gradual decrease period, infection cases due to gatherings increased the number of new confirmed cases, but the trend was decreasing. This period lasted for 20 days, which constituted a concentrated outbreak period for the contact networks of the confirmed cases and cluster infections. By 22 February 2020, the epidemic was under control, which was appropriate for recovering social and economic activities in an orderly manner. Finally, the epidemic arrived at the stabilization stage, with a small number of new confirmed cases and a gradually stabilizing cumulative number of confirmed cases. New confirmed cases in this period were mainly caused by gatherings, but the impact was much smaller than that in the first two periods. For example, 15 new cases were confirmed in a company on 26 February 2020, which were caused by contact with confirmed cases. Although the epidemic situation in the BTH area had stabilized, strict measures against gatherings were still necessary to reduce outbreaks. These measures included home quarantine and strict control of population density (39).

**Figure 3:**
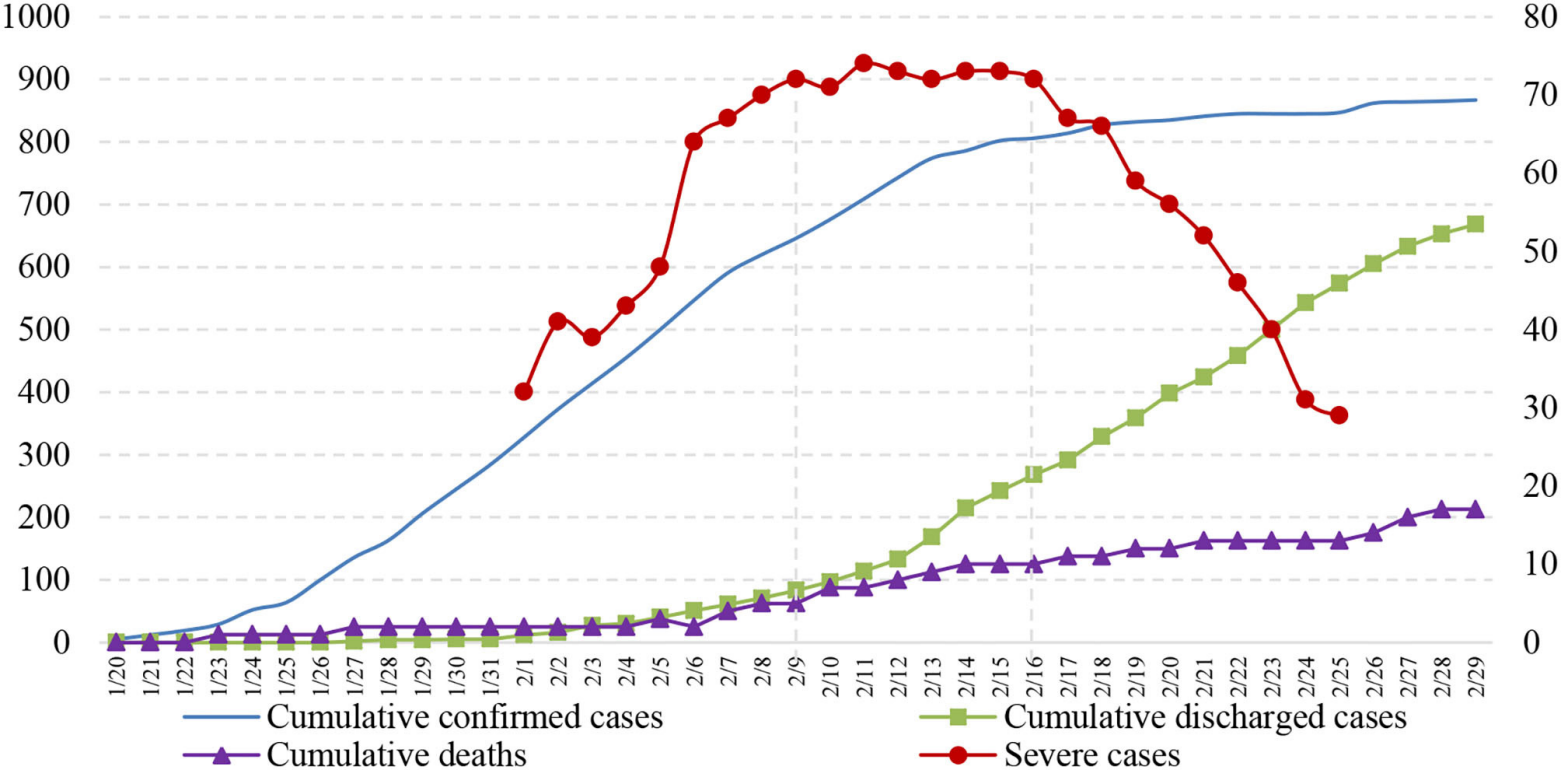
Hospitalization of confirmed cases.

The prevention and control of COVID-19 involve not only a reduction in infector numbers but also the proper treatment of all confirmed cases. The main efficacy indicators for hospital treatment of confirmed cases are discharge rate and mortality rate; thus, successful treatment of severe cases is the key to reducing the mortality rate (38, 41). The number of severe cases among the confirmed cases in the BTH area has been published since 1 February 2020, which provides a reference for understanding the dangers of COVID-19 at the macro level. The number of severe cases in the BTH area showed an increasing, stabilizing, and decreasing trend, as shown in Figure 3. The proper treatment of severe cases benefits from the high-quality medical resources in the BTH area and advanced treatment methods used in China. For example, China implemented the “one infector, one plan” treatment plan for every confirmed case (42). Furthermore, the rate of cumulative discharge is higher than that of cumulative death, indicating that the current treatment methods for confirmed cases have significant clinical effects, and the epidemic prevention and control measures are effective.

### Meso Transmission Pattern: Spatial Pattern of Epidemic Transmission

In this section, using the spatial distribution characteristics of confirmed cases in all 13 cities in the BTH area as well as contact tracing data published by the local Health Commission, we report the analyzed transmission pattern at the meso scale using the composite-group model, which is critical to fulfilling the territorial duty of “preventing the transport of imported cases from outside the region and promoting movement order inside the region.”

**Figure 4:**
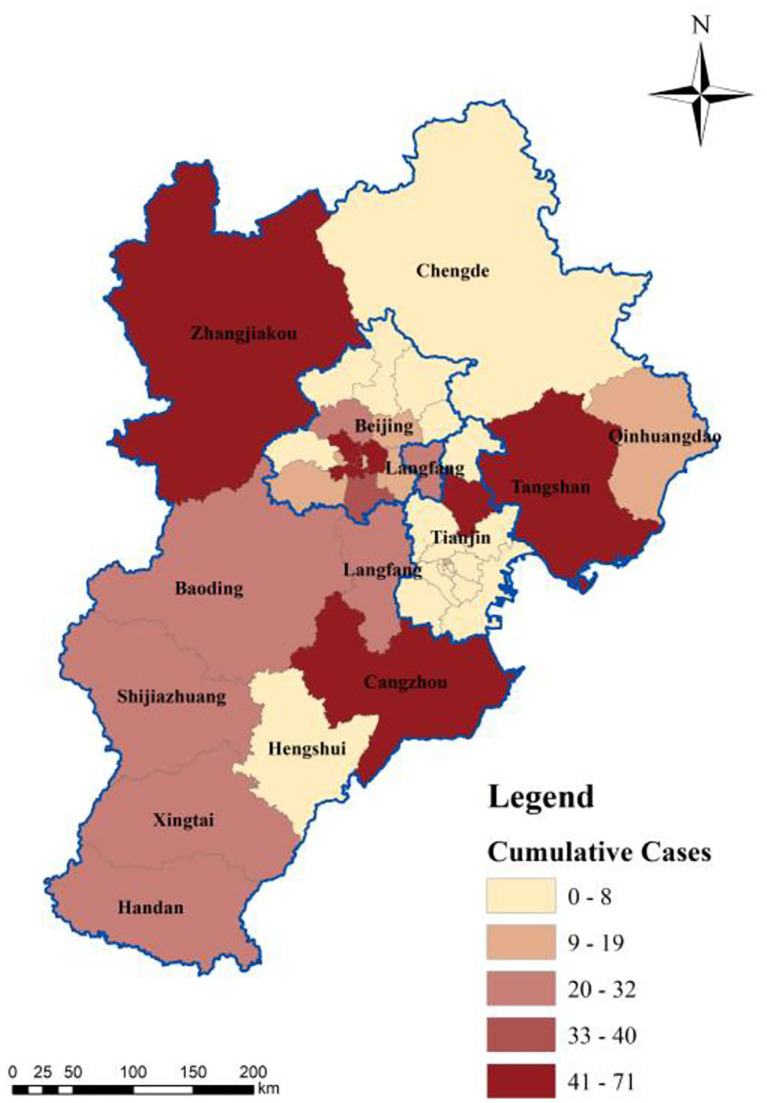
Spatial distribution of confirmed cases.

According to the cumulative number of confirmed cases, the spatial distribution of confirmed cases is mapped. The 13 cities in the BTH area were categorized into most severely affected areas, severely affected areas, and least severely affected areas, as shown in Figure 4.

**Figure 5:**
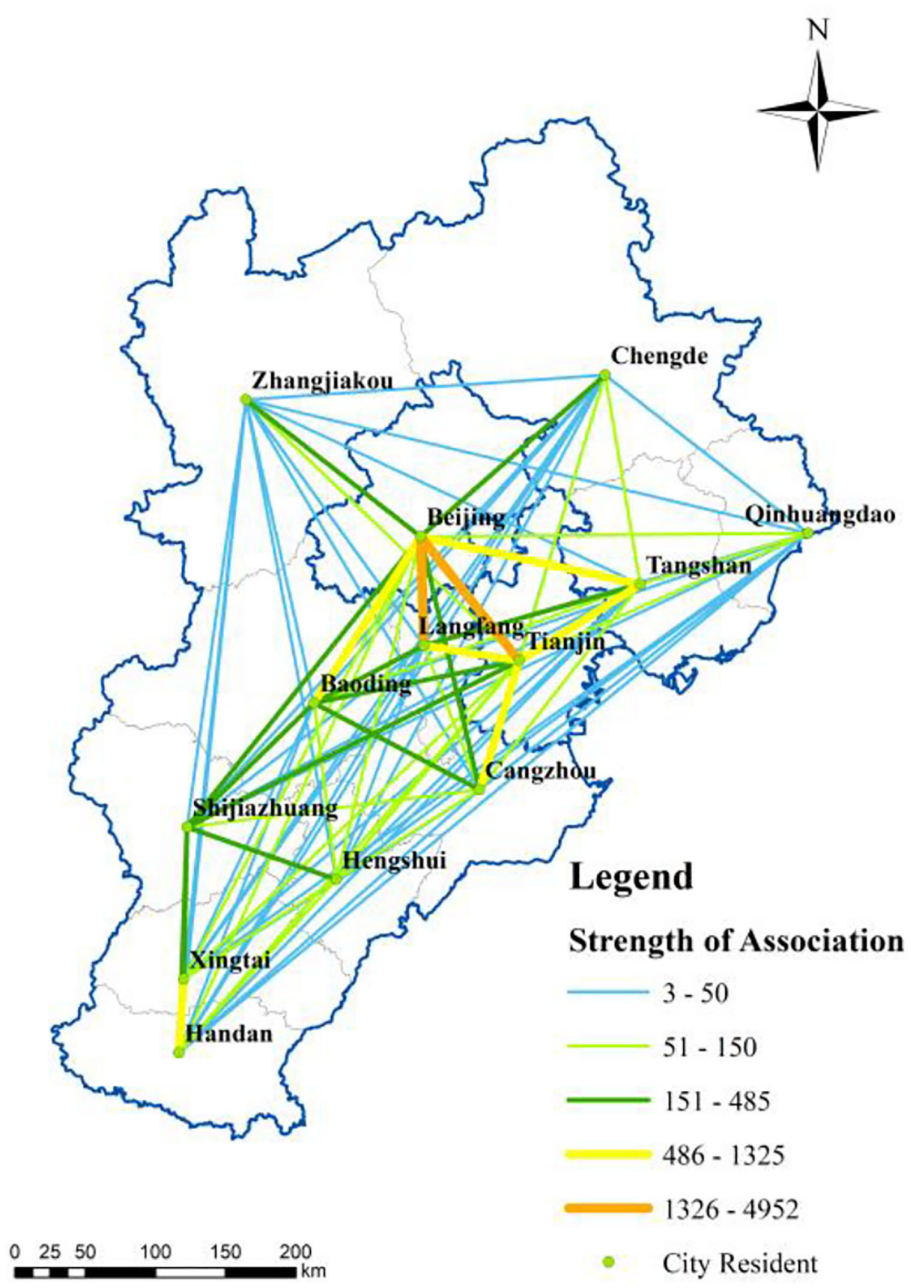
Association intensity among cities.

The most severely affected cities, with more than 40 confirmed cases in total, included Beijing, Tianjin, Zhangjiakou, Tangshan, and Cangzhou. These cities are migration destinations or located at the terminal of the movement network, making them conducive to transport of imported cases and local transmission. Beijing and Tianjin are the centers in the movement network, with high population gathering and transmission risks. However, the prevention and control measures taken in such critical cities were not rigorous enough to effectively screen suspected patients and break down the personal contact network of confirmed cases, leading to a series of outbreaks, such as the shopping mall outbreak in Tianjin and family gathering outbreaks in Zhangjiakou City. Moreover, the horizontal connections between these cities are weak, as some cities only have strong connections with Beijing or Tianjin. For example, Tangshan and Cangzhou have strong connections with Beijing and Tianjin, respectively [Figure 5, data source: (43)].

Second, the severely affected cities (20–39 cumulative confirmed cases) included Handan, Xingtai, Shijiazhuang, Baoding, and Langfang, where the confirmed cases were mainly imported, and local transmission was effectively controlled. These cities hold strong horizontal interconnections with each other (Figure 5), forming the southern corridor at the spatial scale. This corridor is a critical link between the core area in the urban agglomeration and other regions, and thus plays a critical role in transporting confirmed COVID-19 cases. The high population mobility also raises the epidemic transmission risk, especially in airports located in Beijing, Tianjin, and Shijiazhuang, which are important in preventing and controlling epidemics imported from other countries.

Finally, the least severely affected cities (with less than 19 confirmed cases), including Chengde, Qinhuangdao, and Hengshui, have fewer imported cases and lower local transmission than the more severely affected cities. These cities hold single functions in the BTH area; for example, the water conservation site of the BTH area is located in Chengde City. Moreover, their connection with other cities is weak (Figure 5), leading to low pressure on epidemic prevention and control.

**Figure 6:**
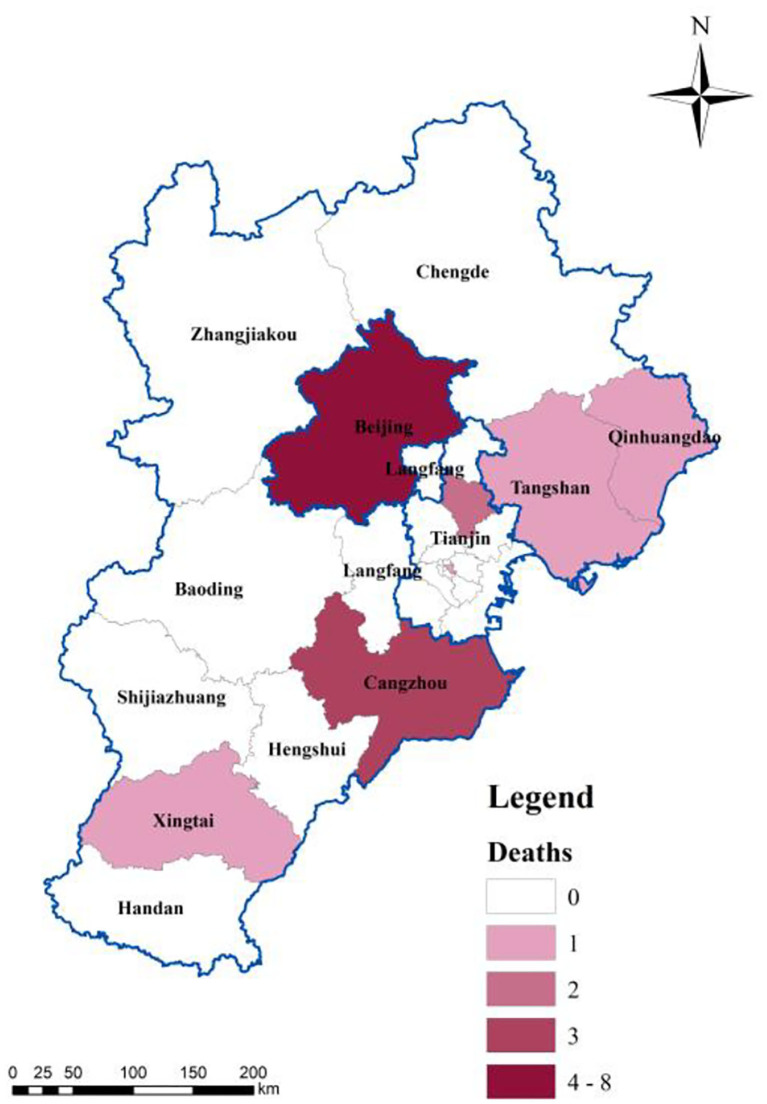
Spatial distribution of deaths.

**Figure 7:**
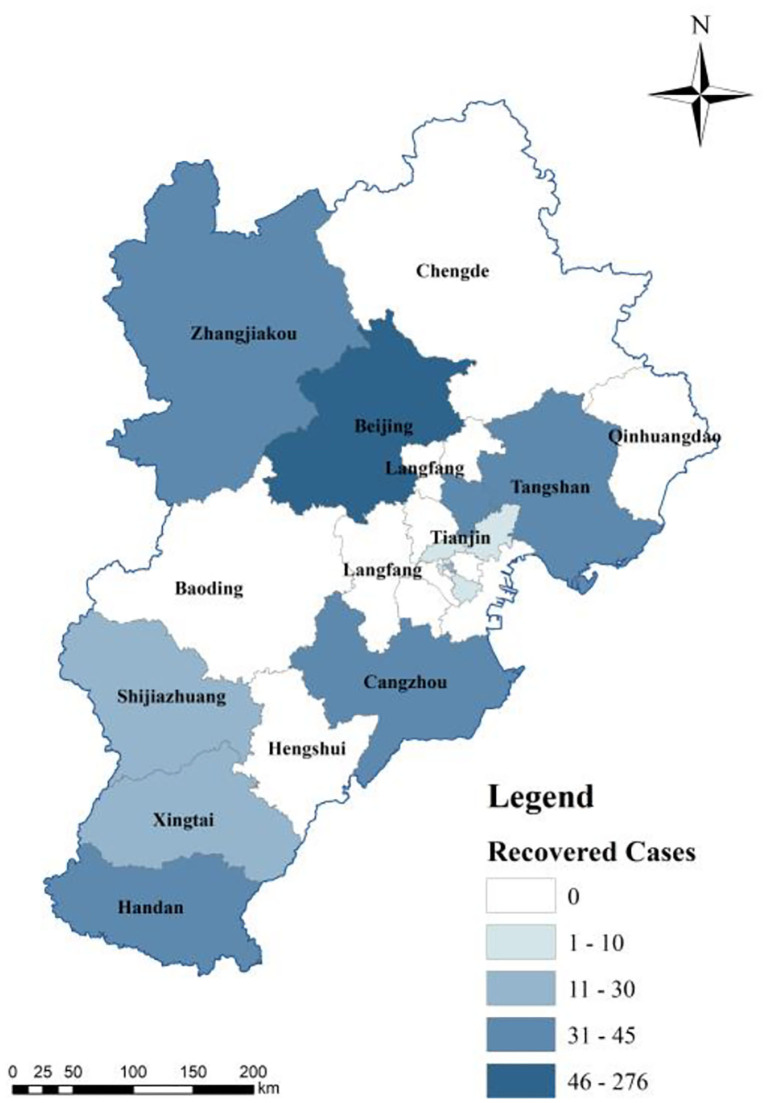
Spatial distribution of discharged cases.

The spatial distribution of hospitalized infectors was determined *via* spatial analysis of discharged cases and deaths to reflect the effectiveness of the current treatment measures. The number of deaths in the BTH area was small. For example, Zhangjiakou City, one of the most severely affected cities, has not reported any death (Figure 6). The deaths in the BTH area are concentrated in Beijing and Cangzhou because of medical resources shared between cities in the BTH area and the free medical treatment services provided for all infectors (44). A large number of discharged cases (Figure 7) indicates that the treatment is effective, even though the proportion of the elderly population is relatively high; in fact, people aged 60 and above account for 25.9% of the cumulative confirmed cases in Beijing. In particular, most of the elderly over 75 years old suffer from serious underlying diseases, making them vulnerable and slow to recover (38). Several cities, such as Baoding and Langfang, discharged all infectors by the end of February, indicating a great success in epidemic prevention and treatment. An important reason for the lower number of discharged cases in the other regions is the lower number of cumulative confirmed cases (Figure 7).

### Micro Transmission Pattern: Contact Characteristics of Confirmed Cases

According to the contact tracing data published by the local government, the first wave of infection in the BTH area was caused by imported cases from Wuhan, Hubei Province. The same trend was observed in other regions of China (21). After the lockdown of Wuhan City, the confirmed cases gradually changed from imported cases to local transmission cases infected through personal contact networks; for example, the proportion of imported cases in Tianjin and Hebei was 26 and 34%, respectively.

**Figure 8:**
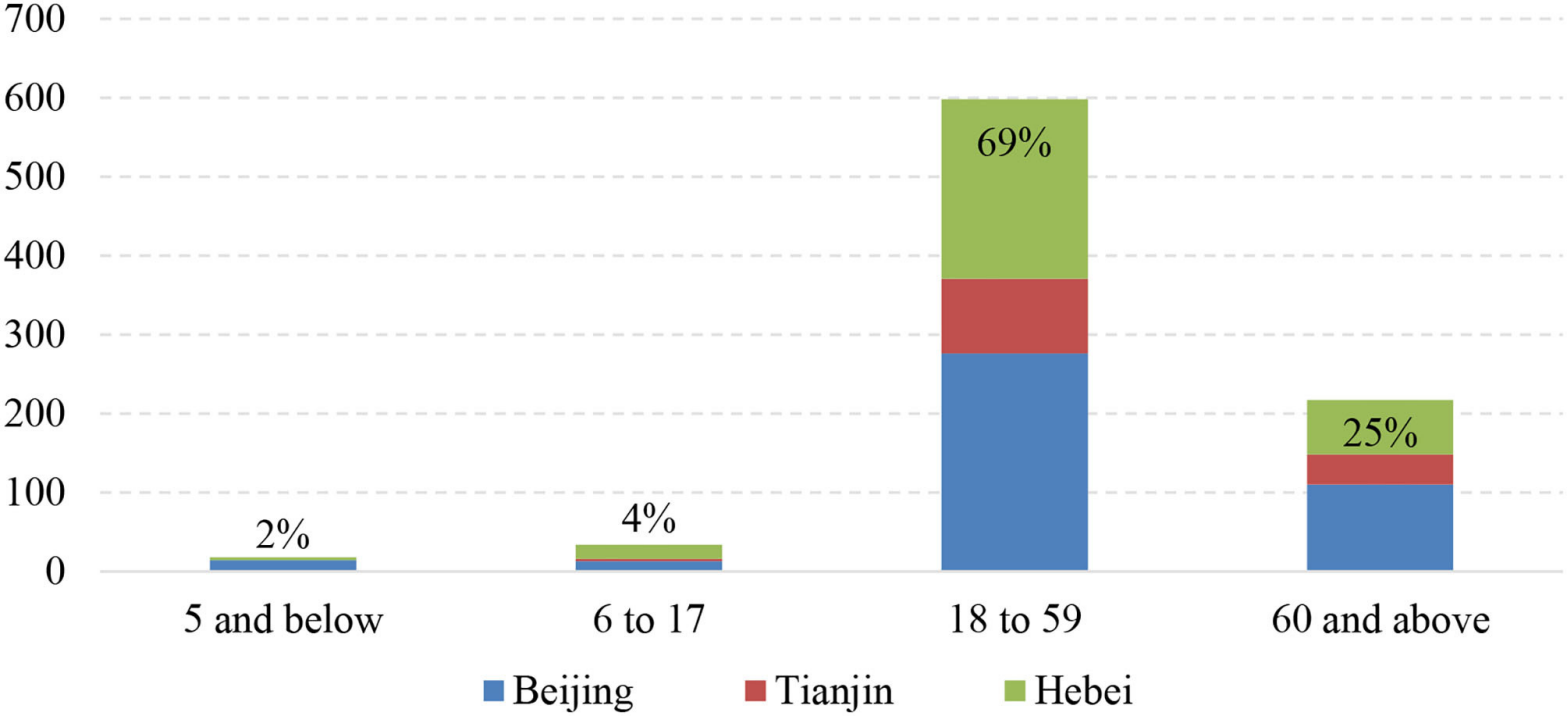
Age groups of confirmed cases in the BTH area.

Static contact network dominates micro transmission. Static contact refers to the direct contact between susceptible persons and infectors in a personal contact network, including colleagues, family members, and carpoolers. Based on the age distribution of confirmed cases (Figure 8), most of the infected cases were young and middle-aged people 18–59 years of age. This is because of their strong willingness to move around and their complex personal contact networks with dynamic boundaries. Although the contact network of the elderly group is limited, the personal contact networks of the young and middle-aged groups tend to overlap with the elderly group during the Spring Festival owing to traditions such as traveling to hometown and visiting relatives and friends during this period.

**Figure 9:**
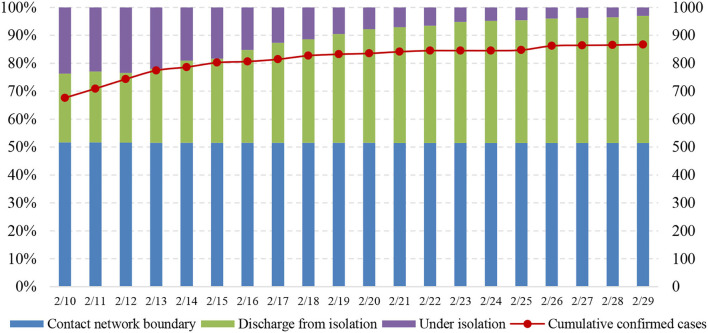
Total number of close contacts by confirmed cases.

Dynamic contact network refers to the boundary of a personal contact network, represented by the total number of close-contact cases, which is shrinking. The defined boundary can be employed to analyze the potential risk of epidemic transmission, as close-contact cases are at a high risk of becoming new confirmed cases, especially in the gradual decrease and stabilization phases of the epidemic. The numbers of close-contact cases and people under isolation have been published since 10 February 2020. The cumulative number of close-contact cases represents the contact network of all confirmed cases, equaling the sum of medical observers who are still in medical isolation and those who have been discharged from medical isolation. As shown in Figure 9, the number of close-contact cases under medical isolation decreased rapidly from 5,543 cases on 13 February to 942 cases on 29 February, during which the cumulative number of confirmed cases stabilized. This indicates that the contact network boundary of confirmed cases is narrowing, and measures on breaking down transmission links are effective to prevent and control the epidemic transmission.

### Comprehensive Risk Assessment of the Epidemic in the BTH Area

Based on the proposed epidemic conceptual model, the comprehensive risk evaluation model was set to the local infection rate (base coefficient, BR), external inflow index (θ), and city movement intensity index (β). The calculation formula is presented as Equation (2), and the index system is explained in Table 1. The data sources were confirmed cases published daily by the local government, the national statistical bulletin on city economic and social development (2019), and Baidu Migration Big Data (http://qianxi.baidu.com/).


(2) $$\begin{array}{l} \text{C}{\text{R}}_{\text{i}}=\text{B}{\text{R}}_{\text{i}}*\theta_{\text{i}}*\beta_{\text{i}} \end{array}$$

**Table 1:** Index system for calculation of comprehensive epidemic risk in the BTH area.

**Dimensions**		**Name**	**Specifications**	**Unit**
Basic coefficient	Macro	Local infection rate	The ratio between the cumulative confirmed cases and total local population	-
Expansion coefficient	Meso	Average external inflow index	The scale of population moving from other cities in mainland China	-
	Micro	Average city movement intensity index	The ratio between the population scale with movement within a city and total local population	-

where CR stands for comprehensive risk, and i (=1,2,3, …,13) represents 13 cities in the BTH area.

**Figure 10:**
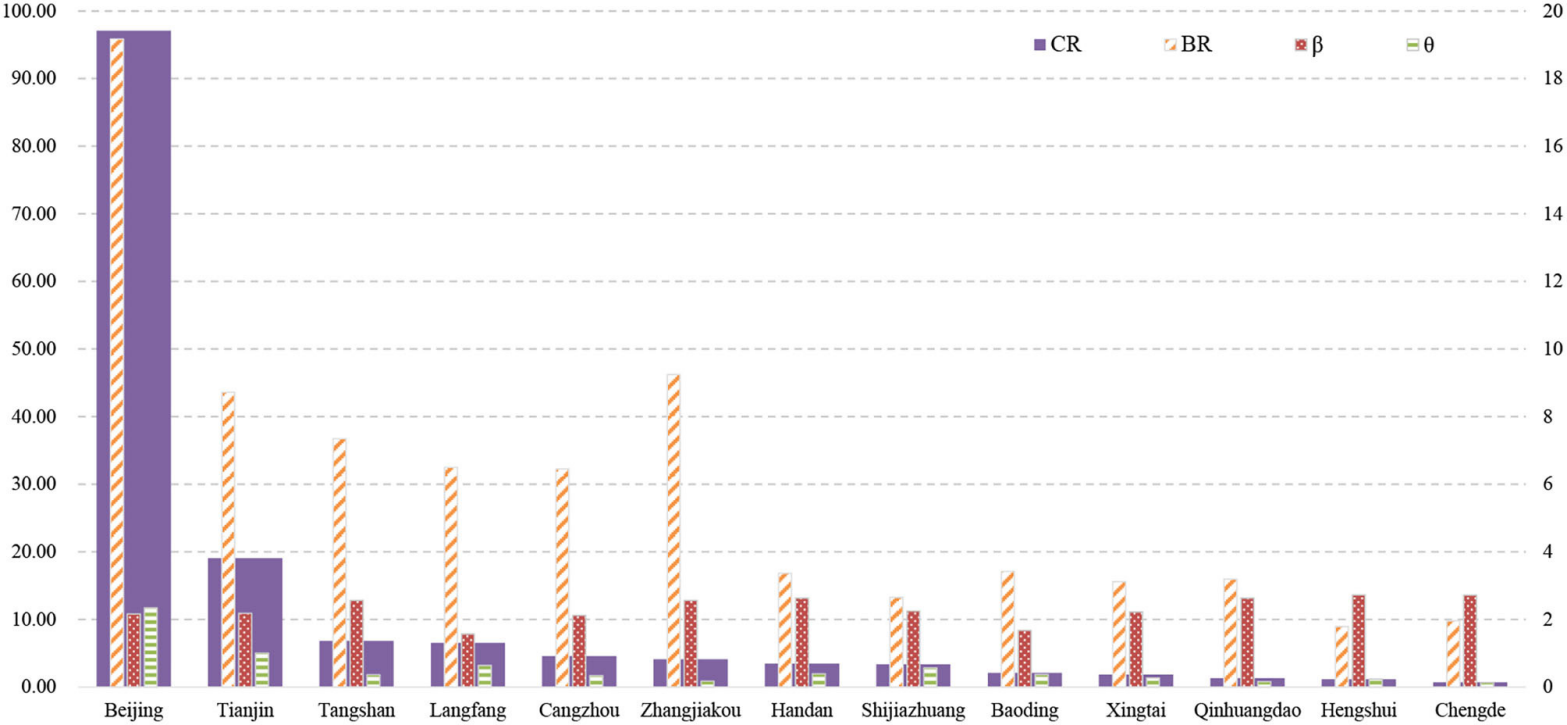
Comprehensive risk of the epidemic in the BTH area.

Using Equation (2) and the built index system, the comprehensive risk of the COVID-19 epidemic in 13 cities in the BTH area was assessed, and the results are presented in Figure 10. Based on the calculated comprehensive risk value, the 13 cities were classified into five groups: high-risk (Beijing), medium-high-risk (Tianjin), medium-risk (Tangshan, Langfang), medium-low-risk (Cangzhou, Zhangjiakou, Handan, and Shijiazhuang), and low-risk (Baoding, Xingtai, Qinhuangdao, Hengshui, and Chengde) cities. The cities with the highest risk included Beijing, Tianjin, Tangshan, and Langfang, which constitute the core region of the BTH area. These four cities have numerous social and economic connections (43). For example, the Capital Steel Group moved to Tangshan from Beijing in 2005 (45), building a strong connection between Beijing and Tangshan. In addition, according to the Baidu Migration Big Data, the inflow population to Beijing and Tianjin is mainly from Langfang and Tangshan.

First, Beijing and Tianjin are high- and medium-high-risk cities, respectively. The high epidemic risk in Beijing and Tianjin is the result of a high BR value and positive expansion coefficient. On one hand, the BR value of Beijing and Tianjin is high because the number of confirmed cases in Beijing (413 cases) and Tianjin (136 cases) are much larger than that in the other 11 cities, which may lead to the high infection rate in Beijing and Tianjin (Figure 10); on the other hand, Beijing and Tianjin are the core cities in the urban agglomeration, which experience large-scale population migrations. This may result in a high migration index, leading to a high comprehensive risk value.

Medium-risk cities, such as Tangshan and Langfang, have relatively high BR values, but their inner-city movement intensity (β) is not much lower than that of Beijing and Tianjin. Owing to the low inflow population (θ) the comprehensive risk value of Tangshan and Langfang was lower than that of Beijing and Tianjin.

Finally, medium-low-risk cities, such as Cangzhou and Zhangjiakou, are severely affected by the epidemic, with more than 40 confirmed cases in total. The basic coefficient of Zhangjiakou is significantly high, ranking second in the BTH area. However, the low inflow population reduces the comprehensive risk of the epidemic. The Zhengding International Airport is located in Shijiazhuang City, and Handan City is the “southern gate” of the urban agglomeration; thus, these two cities have relatively high inflow population, which increases their comprehensive epidemic risk. Moreover, although low-risk cities have high intensity of inner-city movement, the comprehensive risk values are still low owing to their low inflow population.

## Discussions

**Figure 11:**
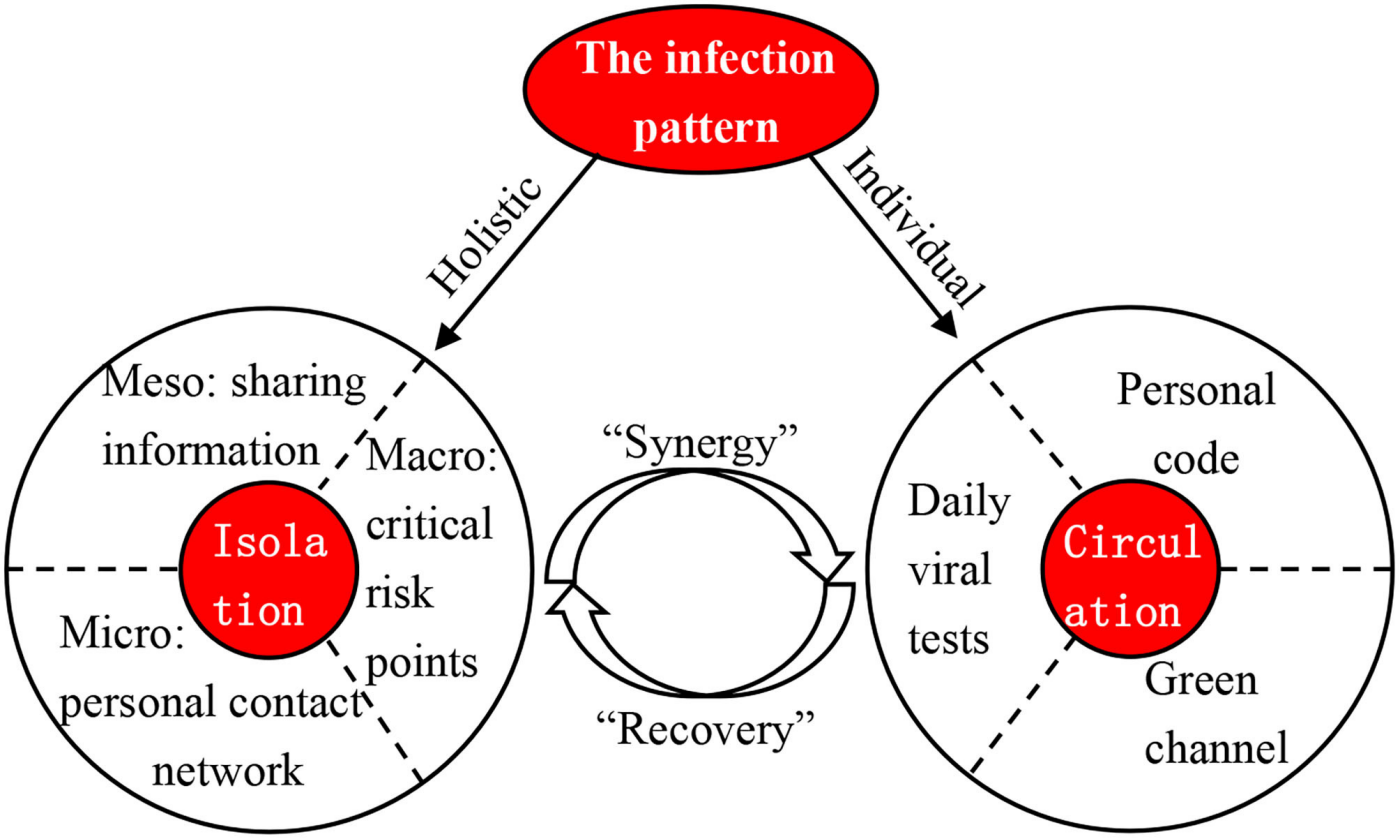
Conceptual framework of the “two-wheels” strategy.

To address the COVID-19 pandemic, isolation is the mainstream in the panic phase, but the core principle is to balance the isolation requirement and circulation demand. This should be guided by the infection and transmission patterns of COVID-19, since more outbreaks in China initially occurred owing to goods-to-person transmission, followed by person-to-person transmission. Based on this information, we mapped a “two-wheels” conceptual framework (Figure 11) to discuss the implications of future measures to address external shocks, such as COVID-19.

In the first wheel, a holistic perspective is necessary to address the external shock (46) because the indirect impacts of the shock are more serious than the direct impacts to some degree. The direct impact of the COVID-19 pandemic is its significant influence on public health as well as social and economic activities (47), and the indirect impacts include crises in public mental health, trust in government policy, food supply, and social cohesion (48, 49). The holistic perspective requires integration at the macro, meso, and micro scales, especially in urban agglomeration. At the macro level, information on the pandemic transmission should be published in a coordinated manner (50), and the transmission characteristics, as well as prevention and control priorities across the region, should be clarified. For example, information on the epidemic at the regional level should be released in the name of the Regional Epidemic Prevention and Control Joint Meeting to identify regional risk points and enhance the circulation confidence inside or outside of the region. The Chinese mobile cabin hospitals are employed at the regional scale to isolate individuals (51), and comprehensive risk evaluation can also provide an insight into the actions to be taken at the macro level. At the meso level, information sharing within and between local governments is a basic requirement for regional protection and the reduction of unintended results (52). This kind of information sharing should exist not only in government agencies but also widely in bus stations, railway stations, and airports, where the inflow population often stays. At the micro level, contact tracing is indispensable to immediately identify the personal contact network of every confirmed case. Both static and dynamic contact networks within the region are required and should be published as early as possible.

In the second wheel, a new normal of living with COVID-19 in the background is going to be achieved (8), as the daily life and economic activities have shifted from free movement in the old normal to coordinated resonance in the new normal. In the new normal period, individual strategy should be implemented, such as the “one city, one case” and “one city, one policy” strategies in China, and the urban governance capacity will play a critical role in the battle against the COVID-19 pandemic (53). This is required not only for scientific prevention and control but also for regional economic resistance and recovery (54). According to the report of the WHO-China Joint Mission on COVID-19 (55), strict isolation and social distancing measures can effectively reduce the local transmission risk after the epidemic has been imported. Regional risk points and regional prevention and control priorities can be identified based on the results of comprehensive epidemic risk assessments. The regional risk points should take daily or weekly viral tests, which function as an indicator to start isolation. Specifically, for high-risk areas (Beijing-Tianjin-Langfang-Tangshan), the key to epidemic prevention and control is limiting large-scale population inflow. For example, online working reduces the inflow population and the scale of gathering in the workplace. Labors and items can enter through the green channel. Moreover, on the inner-city scale, everybody with a health code is permitted to move around within the city, and some key production industries, such as the water and gas supply industry, can ensure their normal operations.

## Conclusions

Urban agglomeration faces various internal and external shocks. The core principle in addressing an external shock is to follow its evolution pattern. For example, in the COVID-19 pandemic, isolation requirements should follow the infection and transmission patterns of the disease. This study analyzed the transmission pattern theoretically and practically in the macro, meso, and micro levels during the panic phase of the pandemic. Initially, the COVID-19 epidemic in the BTH area was under control by the end of February 2020. The period of rapid increase lasted for 14 days, which is the same as the incubation period of COVID-19. The spatial distribution of confirmed cases was closely related to the city connection intensity in the BTH area. The personal contact network could be defined by the scale of the population under medical isolation. Furthermore, the highest risk was present in the core region of the urban agglomeration, namely Beijing, Tianjin, Tangshan, and Langfang. Low-risk cities had the characteristics of low inflow population, but not a low inner-city movement. Finally, both holistic and individual perspectives are critical in addressing external shocks, along with information sharing and stakeholder involvement. In the future, exploring the governance structure in the panic phase is still necessary to address external shocks.

## Data Availability Statement

Publicly dataset and policies were analyzed in this study. This data can be found here: http://wjw.beijing.gov.cn/wjwh/ztzl/xxgzbd/; http://wsjk.tj.gov.cn/ZTZL1/ZTZL750/YQFKZL9424/; http://www.hebwsjs.gov.cn/html/yqtb/; http://qianxi.baidu.com/. Policies can be found here: http://www.nhc.gov.cn/xcs/xxgzbd/gzbd_index.shtml; http://www.gov.cn/zhengce/zhengcewenjianku/index.htm. Further inquiries can be directed to the corresponding author/s.

## Author Contributions

DH designed and wrote the manuscript. FW participated in the data collection, the discussion section, and as the corresponding author. SL drew some of the figures and conducted the calculation. All authors contributed to the article and approved the submitted version.

## Funding

This work was supported by the National Natural Science Foundation of China (Grant Nos. 72104018 and 72174219), and the Fundamental Research Funds for the Beijing University of Civil Engineering and Architecture (Grant No. X20041).

## Conflict of Interest

The authors declare that the research was conducted in the absence of any commercial or financial relationships that could be construed as a potential conflict of interest.

## Publisher's Note

All claims expressed in this article are solely those of the authors and do not necessarily represent those of their affiliated organizations, or those of the publisher, the editors and the reviewers. Any product that may be evaluated in this article, or claim that may be made by its manufacturer, is not guaranteed or endorsed by the publisher.
